# Quantitative genomics of starvation stress resistance in *Drosophila*

**DOI:** 10.1186/gb-2005-6-4-r36

**Published:** 2005-03-24

**Authors:** Susan T Harbison, Sherman Chang, Kim P Kamdar, Trudy FC Mackay

**Affiliations:** 1 Department of Genetics, North Carolina State University, Raleigh, NC 27695, USA; 2 WM Keck Center for Behavioral Biology, North Carolina State University, Raleigh, NC 27695, USA; 3 The Torrey Mesa Research Institute, 3115 Merryfield Row, San Diego, CA 92121, USA; 4 Current address: Department of Neuroscience, University of Pennsylvania Medical School, Philadelphia, PA 19104, USA

## Abstract

**Background:**

A major challenge of modern biology is to understand the networks of interacting genes regulating complex traits, and the subset of these genes that affect naturally occurring quantitative genetic variation. Previously, we used *P*-element mutagenesis and quantitative trait locus (QTL) mapping in *Drosophila *to identify candidate genes affecting resistance to starvation stress, and variation in resistance to starvation stress between the Oregon-R (Ore) and 2b strains. Here, we tested the efficacy of whole-genome transcriptional profiling for identifying genes affecting starvation stress resistance.

**Results:**

We evaluated whole-genome transcript abundance for males and females of Ore, 2b, and four recombinant inbred lines derived from them, under control and starved conditions. There were significant differences in transcript abundance between the sexes for nearly 50% of the genome, while the transcriptional response to starvation stress involved approximately 25% of the genome. Nearly 50% of *P*-element insertions in 160 genes with altered transcript abundance during starvation stress had mutational effects on starvation tolerance. Approximately 5% of the genome exhibited genetic variation in transcript abundance, which was largely attributable to regulation by unlinked genes. Genes exhibiting variation in transcript abundance among lines did not cluster within starvation resistance QTLs, and none of the candidate genes affecting variation in starvation resistance between Ore and 2b exhibited significant differences in transcript abundance between lines.

**Conclusions:**

Expression profiling is a powerful method for identifying networks of pleiotropic genes regulating complex traits, but the relationship between variation in transcript abundance among lines used to map QTLs and genes affecting variation in quantitative traits is complicated.

## Abstract 2

The efficacy of transcriptional profiling for identifying networks of pleiotropic genes regulating complex traits was assessed. The transcriptional response to starvation stress in males and females of the Oregon-R and 2b *Drosophila* strains, as well as four recombinant inbred lines derived from them, was shown to be different between the sexes and to involve approximately 25% of the genome.

## Background

Quantitative traits affecting morphology, physiology, behavior, disease susceptibility and reproductive fitness are controlled by multiple interacting genes whose effects are conditional on the genetic, sexual and external environments [1]. Advances in medicine, agriculture, and an understanding of adaptive evolution depend on discovering the genes that regulate these complex traits, and determining the genetic and molecular properties of alleles at loci that cause segregating genetic variation in natural populations. Assessing subtle effects of induced mutations on quantitative trait phenotypes in model organisms is a straightforward approach to identify genes regulating complex traits [1-3]. However, the large number of potential mutations to evaluate, the necessity to induce mutations in a common inbred background, and the level of replication required to detect subtle effects [1] all limit the feasibility of systematic whole-genome mutagenesis screens for complex traits in higher eukaryotes. Mapping quantitative trait loci (QTLs) affecting variation in complex traits to broad genomic regions by linkage to polymorphic molecular markers is also straightforward. However, our ability to determine what genes in the QTL regions cause the trait variation is hampered by the large number of recombinants required for high-resolution mapping, and the small and environmentally sensitive effects of QTL alleles [1,4].

There has been great excitement recently about the utility of whole-genome transcriptional profiling to identify candidate genes regulating complex traits, by assessing changes in gene expression in the background of single mutations affecting the trait [5,6], between lines selected for different phenotypic values of the trait [7], and in response to environmental stress and aging [8-12]. Transcript abundance is also a quantitative trait for which there is considerable variation between wild-type strains [11,13-17], and for which expression QTLs (eQTLs) [18] have been mapped [15-17,19]. Thus, candidate genes affecting variation in quantitative trait phenotypes are those for which the map positions of trait QTL and eQTL coincide [16,20].

Transcript profiling typically implicates hundreds to thousands of genes in the regulation of quantitative traits and associated with trait variation between strains; the majority of these genes are computationally predicted genes that have not been experimentally verified. To what extent do changes in transcript abundance predicate effects of induced mutations and allelic variants between strains on quantitative trait phenotypes? It is encouraging that several studies have confirmed the phenotypic effects of mutations in genes implicated by changes in expression [5-7]. However, limited numbers of genes were tested, and their choice was not unbiased. None of the candidate QTLs nominated by transcriptional profiling has been validated according to the rigorous standards necessary to prove that any candidate gene corresponds to a QTL [1,4]. To begin to answer this question, we need to compare gene-expression data with genes known to affect the trait from independent mutagenesis and QTL mapping studies. This comparison has not been possible to date because there are only a few complex traits for which the genetic architecture is known at this level of detail, one of which is resistance to starvation stress in *Drosophila*.

Previously, we used *P*-element mutagenesis in an isogenic background to identify 383 candidate genes affecting starvation tolerance in *D. melanogaster *[21]. Further, we mapped QTLs affecting variation in starvation resistance between two isogenic *Drosophila *strains, Oregon-R (Ore) and 2b [21], followed by complementation tests to mutations to identify twelve candidate genes affecting variation in starvation resistance between these strains [21]. Here, we used Affymetrix *Drosophila *GeneChips to examine expression profiles of two starvation-resistant and two starvation-sensitive recombinant inbred (RI) lines, as well as parental lines Ore and 2b, under normal and starvation stress conditions. We used a statistically rigorous analysis to identify genes whose expression was altered between the sexes, during starvation stress treatment, between lines, and interactions between these main effects. In the comparison of expression profiling with the *P*-element mutagenesis performed previously, we found nearly 50% concordance between the effects of 160 *P*-element mutations on starvation stress resistance and changes in gene expression during starvation - 77 mutations with significant effects also had significant changes in transcript abundance, while 83 mutations did not affect the starvation resistance phenotype, yet had significant changes in transcript level. We identified 153 novel candidate genes for which there was variation in gene expression between the lines and which co-localized with starvation resistance QTLs. However, we did not detect genetic variation in expression for any of the candidate genes identified by complementation tests. Our efforts to associate genetic variation in expression with variation in quantitative trait phenotypes is confounded by the observation of widespread regulation of transcript abundance by unlinked genes, the difficulty in detecting rare transcripts that may be expressed in only a few cell types at a particular period of development, and genetic variation between QTL alleles that is not regulated at the level of transcription.

## Results

### The sexually dimorphic transcriptome

Nearly one-half of the genome (6,569 probe sets) exhibited significantly different transcript levels between the sexes (*P*(Sex) < 0.001), with 3,965 probe sets upregulated in females and 2,604 probe sets upregulated in males (the complete list is given in Additional data file 1). The greatest differences in transcript abundance between the sexes were for probe sets implicated in sex-specific functions: chorion, vitelline membrane, and yolk proteins involved in egg production were upregulated in females; and accessory gland peptides, male-specific RNAs, and protein ejaculatory bulb components were upregulated in males. However, the probe sets exhibiting sex dimorphism in expression fell into 28 biological process and 41 molecular function Gene Ontology (GO) categories; for most of these categories, differences in expression between the sexes was unexpected. We determined which GO categories contained significantly different numbers of upregulated probe sets in males and females (Table 1). Genes involved in the biological process categories of cell communication, cell growth and/or maintenance, development, and cell death were upregulated more often in females than in males. Genes involved in the molecular function categories of binding, most enzymes, signal transduction, structural molecules, and regulation of transcription and translation were upregulated in females more often than in males; however, genes encoding oxidoreductase enzymes, carrier transporters and ion transporters were upregulated in males more often than in females (Table 1).

**Figure 1:**
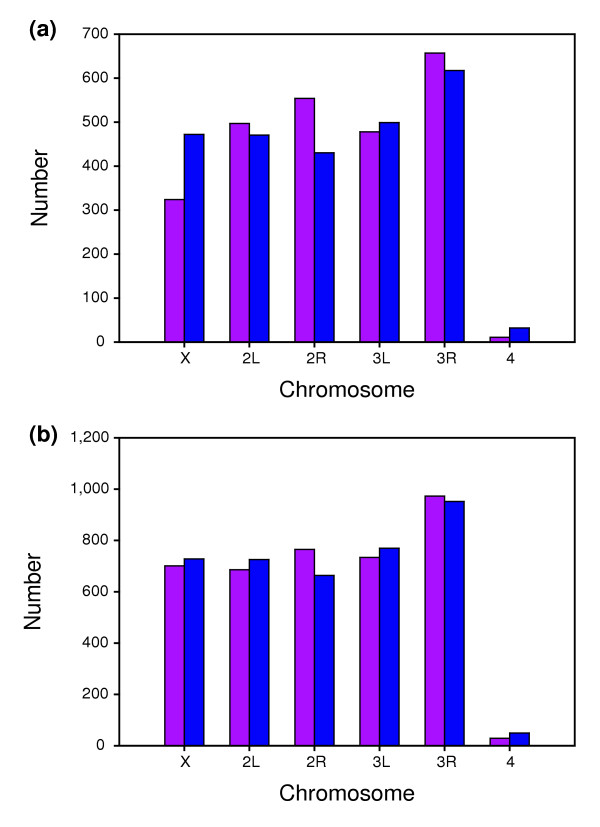
Chromosome locations of genes differentially expressed by sex. **(a) **Observed (magenta) and expected (blue) number of probe sets upregulated in males. **(b) **Observed (magenta) and expected (blue) numbers of probe sets upregulated in females.

The genomic distribution of sex-biased genes was not random (Figure 1). There was a paucity of male-biased genes on the *X *and fourth chromosomes, and an excess on chromosome *2R *(χ^2^_5 _= 100.77; *P *< 0.0001). There was a deficit of female-biased genes on chromosome 4, and an excess on chromosome *2R*(χ^2^_5 _= 29.18; *P *< 0.0001).

### Transcriptional response to starvation stress

We found 3,451 probe sets with significantly different mean transcript levels between the control and starved conditions (*P*(treatment) < 0.001): 1,736 were downregulated (some by as much as 40-fold) and 1,715 were upregulated (at most by 7.2-fold) during starvation (the complete list is available as Additional data file 2). These probe sets fell into 24 biological process and 25 molecular function GO categories. We determined which GO categories had a significantly different number of up- and downregulated probe sets in response to starvation stress. Genes affecting the biological processes of protein and nucleic-acid metabolism (protein biosynthesis; protein catabolism, folding, localization, modification, and repair; biosynthesis of nucleic acid macromolecules and lipids) were upregulated during starvation (Table 2). The expression of genes in three molecular function categories (nucleotide binding, hydrolases binding to acid anhydrides, and ribosome structure) increased during starvation; while defense/immunity proteins, peptidases, cuticle structural proteins, and carrier transport proteins were downregulated (Table 2).

**Figure 2:**
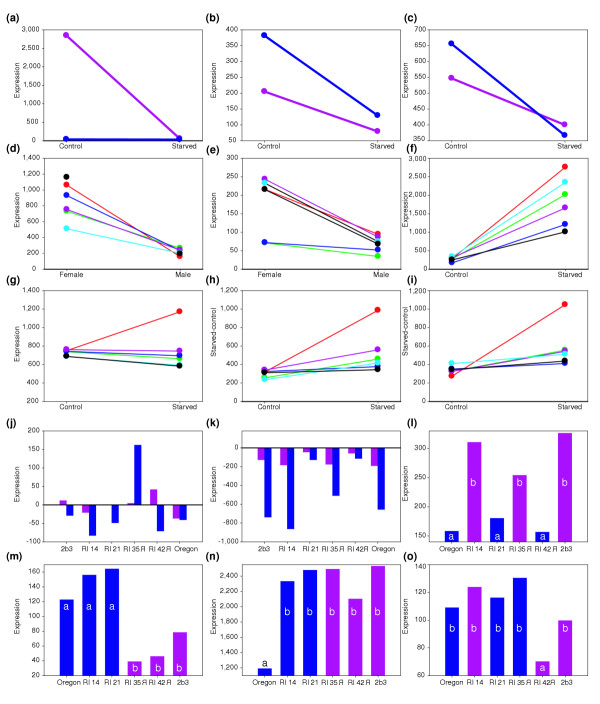
Genetic architecture of transcription. **(a-c) **Sex × treatment interaction for females (magenta)and males (blue): (a) *Chorion protein 38*; (b) *Alkaline phosphatase 4*; (c) *Phosphogluconate dehydrogenase*. **(d-k) **Interactions with line. Ore (black), 2b (red), RI 14 (green), RI 21 (dark blue), RI 35(magenta), RI 42 (light blue). (d, e) Sex × line interaction, averaged over treatments: (d) *modulo*; (e) *l(2) giant larvae*. (f-i) line × treatment interaction, averaged over sex: (f) *CG11089*; (g) *Nervana 1*; (h) *Cyp9b2*; (i) *Peroxiredoxin 2540*. (j, k) Sex × line × treatment interaction. The difference in expression between the starved and control treatments is plotted for females (magenta) and males (blue): (j) *sallimus*; (k) *Esterase 6*. **(l-o) **Regulation of transcript abundance. The same letters denote expression levels that are not significantly different. Magenta indicates 2b and blue indicates Ore genome. (l, m) Linked regulation of variation in transcript abundance: (l) *UDP-glycosyltransferase 35b*; (m) *Signal recognition particle receptor b*. (n, o) Unlinked regulation of variation in transcript abundance: (n) *Arrestin 2*; (o) *Klarsicht*.

The treatment × sex interaction term was significant (*P *< 0.001) for 817 probe sets, of which 715 had significant treatment effects for one or both sexes in the separate sex analyses (Additional data file 3). We categorized these 715 probe sets as sex-specific if significant expression changes in response to starvation occurred in one sex only; as sex-biased if expression levels changed in the same direction in both sexes, but were of different magnitude; or as sex-antagonistic if expression levels significantly changed in both sexes, but in opposite directions (Figure 2a-c). Most probe sets exhibited sex-specific or sex-biased expression, with only two genes, *CG14095 *and *Rpd3*, meeting the sex-antagonistic criterion. More probe sets exhibiting sex-specific or sex-biased expression were downregulated (454) than upregulated (263) during starvation. Starvation stress was accompanied by reduced expression of genes involved in the developmental processes of gametogenesis and sex determination as well as signal transduction in females, and of genes involved in mechanosensory and reproductive behavior in males (Table 2).

### Transcript abundance versus mutations

The genes represented by probe sets with significant treatment and/or treatment × sex effects are candidate genes for starvation resistance. Previously, we screened 933 co-isogenic single *P*-element insertion lines for their effect on starvation resistance [21]. Of these insertions, 383 had significant effects on starvation resistance, while the remaining 550 did not [21]. Of the 933 lines, we know the locations of the 385 of the inserts and that genes tagged by these inserts are represented on the array. Thus, we can directly compare the extent to which effects of *P*-element mutations on the starvation phenotype correspond to changes in transcript abundance in response to starvation. This comparison allows us to assess the hypothesis that changes in transcript abundance can be used to identify candidate genes with effects on phenotype, an hypothesis implicit in previous microarray studies [5-7]. Overall, there was no statistical association between the phenotypic and transcript data (χ^2^_1 _= 0.0006, *P *= 1). For 194 genes, there was agreement between the phenotype and the expression level. Seventy-seven genes had significant differences in both transcript profile and mutant phenotypes, and 117 genes affected neither phenotype nor expression level (Additional data file 4). There was disagreement between the expression and phenotypic analyses for 191 genes (49.6%): 108 of the genes tagged by *P*-elements affected starvation resistance, but did not display differences in transcript level in response to starvation stress, and *P*-element insertions in 83 genes that exhibited significant differences in transcription in response to starvation did not have significant phenotypic effects on starvation tolerance (Additional data file 4).

### The genetic architecture of transcription

A total of 706 probe sets exhibited variation in expression among the six lines; 640 probe sets were significant (*P *< 0.001) for the main effect of line, 190 for the line × sex interaction, 200 for the line × treatment interaction, and 85 for the three-way interaction of line × sex × treatment (Additional data file 5, and Figure 2d-k). Thus, transcript abundance exhibits both genotype by sex and genotype by environment interaction.

We used *post-hoc *Tukey tests to group lines with similar levels of gene expression, and compared the expression clusters with the Ore and 2b genotype of the six lines. There are three possible scenarios by which genetic variation in transcript abundance could arise. First, genetic variation in regulatory regions of gene *A *causes variation in the expression of gene *A *(*cis*-acting regulatory variation). Second, genetic variation in regulation of gene *B *causes variation in expression of *A*, which is itself not genetically variable (*trans*-acting regulatory variation). Third, genetic variation in both gene *A *and gene *B *affect the transcript abundance of gene *A *(*cis*- and *trans*-acting regulatory variation). These two-locus interactions could be additive or epistatic. We observe whether or not expression of gene *A *co-segregates with markers differentiating the two parental strains. Co-segregation will always be observed in case 1. It could also be observed in cases 2 and 3 if gene *B *is tightly linked to gene *A*, such that it is not separated by recombination from *A *in the genotypes tested. However, co-segregation will not be observed if gene *A *and gene *B *are unlinked. The most prevalent observation was regulation of expression by unlinked genes. For example, there were unambiguous interpretations for 246 probe sets that were significant for the main effect of line only: 65 (26.4%) were regulated by linked genes and 181 (73.5%) were regulated by unlinked genes (Additional data file 6, and Figure 2l-o). We also inferred linkage of genes regulating expression levels under control and starved conditions separately. There were unambiguous Tukey interpretations for 277 probe sets under control conditions, of which 32 exhibited linked regulatory variation (11.6%) and 245 were regulated by variation at unlinked genes (88.4%). For 244 probe sets under starved conditions, 46 were regulated by polymorphism at linked loci, (18.9%) and 198 were regulated by variation at unlinked genes (81.1%) (Additional data file 7).

### Association of genetic variance in transcription with QTLs

Probe sets from the three-way ANOVA that are significant for the main effect of line and/or line × sex (*P *< 0.001), but not significant for the line × treatment interaction terms, exhibit genetic variation in transcription among the six lines that is independent of the starvation treatment. A total of 489 probe sets met these criteria, and we know the cytological locations of 475 of the corresponding genes. Previously, RI lines derived from Ore and 2b have been used to map QTL affecting variation in life span [22-25], sensory bristle numbers [26], ovariole number [27], courtship signal [28], olfactory behavior [29], metabolism and flight [30], as well as starvation resistance [21]. Genes that exhibit significant differences for the main effect of line and/or line × sex which are located within QTL regions are putative candidate genes corresponding to the QTL [16,20]. We identified several novel putative candidate genes affecting these traits (Additional data file 5). We examined whether probe sets with significant line and/or line × sex effects tended to cluster within regions containing QTL mapped under standard culture conditions, as would be the case if QTL regions were enriched for genes exhibiting transcriptional variation between the parental lines. We found no evidence for such clustering; indeed, the only trait showing a non-random association of probe sets with QTL that survived a Bonferroni correction for multiple tests was in the direction of a deficiency of probe sets in the QTL region (Table 3).

The 217 probe sets with significant line × treatment and/or line × treatment × sex terms (Additional data file 5) represent genetic differences among the lines in response to the starvation treatment. Are these probe sets enriched in regions to which starvation resistance QTL map? We found that 47 of the probe sets meeting these criteria, representing 45 unique genes, fell within starvation resistance QTL regions; and the remaining 170 probe sets, representing 169 unique genes, fell outside the QTL intervals. These probe sets were not over-represented within starvation resistance QTL (χ^2^_1 _= 0.26, *P *> 0.05).

There is significant variation in starvation half-life among the six lines (*P *< 0.0001; Additional data file 8). For those probe sets previously identified as having significant differences in transcript level among the lines, we assessed the extent to which variation in transcript abundance was associated with variation in starvation half-life. We found 281 probe sets with significant correlations (*P *< 0.05) between starvation phenotype and transcript level, for 273 of which the cytological location was known (Additional data file 5). However, 66 of the probe sets associated with starvation half-life mapped to starvation resistance QTL, and 207 did not. Again, these probe sets were not over-represented within starvation resistance QTL (χ^2^_1 _= 0.45, *P *> 0.05).

Although there is no tendency for genes exhibiting variation in transcript abundance among lines to cluster within starvation resistance QTLs, those that do co-localize with the QTLs are candidate genes affecting variation in starvation tolerance between Ore and 2b. We found 155 probe sets, corresponding to 153 candidate genes, which met one or more of the above criteria (Additional data file 5). Most (114, 75%) were predicted genes. The remaining genes (Table 4) are reasonable candidates for starvation resistance QTLs, affecting the processes of protein metabolism, defense/immune response, proteolysis and peptidolysis, and transport.

Complementation tests to mutations have implicated several candidate genes affecting variation between Ore and 2b in olfactory behavior [29] (*Vanaso*), longevity [31,32] (*Dopa decarboxylase*, *shuttle craft *and *ms(2)35Ci*) and starvation resistance [21] (*spalt major*, *Ryanodine receptor 44F*, *crooked legs*, *NaCP60E*, *Phosphoglucose isomerase*, *bellwether*, *numb*, *Punch*, *l(2)rG270*, *l(2)k17002, l(2)k00611*, and *l(2)k03205*). None of these genes exhibited significant differences in transcript abundance between lines.

## Discussion

### The sexually dimorphic transcriptome

Consistent with previous reports [5,11,33,34], we observed highly significant differences in transcript abundance between males and females for nearly half the genome. These differences in transcriptional profiles were not confined to stereotypical sex-specific biological processes. Female transcript levels were upregulated for genes involved in protein biosynthesis, metabolism, and transcription regulation, while male transcript levels were higher for probe sets involved in ion and carrier transporters, as in a previous study of sex differences in transcription in *Drosophila *heads [5]. Differences in transcript abundance between the sexes may be an underlying mechanism for commonly observed sex-specific effects of QTLs associated with a variety of complex traits in *Drosophila *[21-26,32,35,36] and other organisms [37]. Males and females are effectively different environments in which genes act. The chromosomal locations of genes with sex-dependent expression were non-random. We confirmed the apparently general phenomenon that the *Drosophila X *chromosome is depauperate for genes that are upregulated in males [33,34]; *X*-chromosome demasculinization is perhaps attributable to selection against genes that are advantageous in males but deleterious to females [33]. In contrast to previous studies, we observed that chromosome *2R *harbored an excess, and chromosome *4 *a deficiency, of genes that were upregulated in both males and females.

### Transcriptional response to starvation stress

The transcriptional response to starvation stress involved approximately 25% of the genome. The stress profile indicates upregulation of genes involved in growth and maintenance processes and protein biosynthesis, with increased transcription of genes encoding translation initiation and elongation factors, mitochondrial and cytosolic ribosomal structural proteins, and hydrolases involving acid anhydrides. This increase in protein biosynthesis and hydrolase activity can be interpreted as an attempt to use available proteins for nourishment. A similar phenomenon has been observed in the response of yeast [38] and mammalian cells [39] to starvation, where substantial protein and organelle degradation provides substrate to starving cells [40]. Our observation that peptidases, which catalyze the hydrolysis of peptide bonds, were significantly downregulated in response to starvation, is consistent with the preservation of nascent protein chains. The downregulation of carrier activity and defense/immunity proteins indicates that transport across cell membranes slows and the immune response is compromised in starving flies.

We compared our results to those of a previous microarray study investigating gene-expression changes in starved larvae [41]. We found 21 probe sets that were significantly altered in both studies during starvation. Many of these genes have predicted functions that have not been verified experimentally; however, a few of the genes have known functions. *Insulin-like Receptor*, *Serine pyruvate aminotransferase*, *Amylase distal*, and *mitochondrial carnitine palmitoyltransferase I*, genes known to be involved in metabolism, were common to the two studies. Interestingly, *Peroxidasin*, a gene involved in oxygen and reactive oxygen species metabolism was upregulated fourfold in larvae, while it was downregulated 1.61-fold in our study.

Starvation stress was accompanied by reduced expression of genes affecting gametogenesis, by as much as 66-fold in starved female flies. Egg components such as chorion, yolk, and vitelline membrane proteins were among the most severely restricted transcripts, implicating suppression of female reproductive function during starvation. This depressed reproductive function is not unique to flies, as female mice on a calorically restricted diet experience a cessation in estrous cycle [42] and amenorrhea is one of the hallmarks of anorexia nervosa in human females [43]. Several male accessory gland proteins were also downregulated by as much as 6.5-fold during starvation stress. Oddly, six genes affecting spermatogenesis had significantly different levels of transcript abundance between the control and starved flies in both males and females; we found no male-specific differences in transcript abundance for genes involved in spermatogenesis (Additional data files 1 and 2).

Transcription of *Rpd3 *and *CG14095 *was upregulated in females and downregulated in males during starvation. *Rpd3 *is a transcriptional co-repressor, while the function of *CG14095 *is unknown. Sex-antagonistic patterns of expression have been observed in liver tissue studies of ethanol-fed rats [44], suggesting that these expression patterns may not be unique to flies.

The large number of transcripts altered during starvation implies massive pleiotropy; even more so when our conservative significance threshold is taken into account. This is consistent with our previous observation that 383 of 933 single *P*-element insertion lines tested (41%) had direct effects on starvation tolerance [21]. Further, candidate genes identified from the *P*-element screen and from complementation tests of QTL alleles to mutations at positional candidate genes are pleiotropic, and affect cell fate specification, cell proliferation, oogenesis, metabolism, and feeding behaviors [21].

### Transcript abundance versus mutations

To what extent do candidate genes affecting response to starvation stress identified from changes in transcript abundance coincide with those implicated by assessing quantitative effects of *P*-element insertions on starvation tolerance? The resounding lack of an overall statistical association between the two methods is somewhat deceptive. While there was no association overall, if we had only tested the 160 *P*-element mutations corresponding to genes with altered transcript abundance during starvation, we would have found that 77 (48%) actually had phenotypic effects on starvation resistance. The lack of association was caused by 108 genes tagged by *P*-elements that affected starvation resistance, but did not display differences in transcript level in response to starvation stress, and *P*-element insertions in 83 genes that exhibited significant differences in transcription in response to starvation but did not have significant phenotypic effects on starvation tolerance. Genes affecting starvation that are regulated post-transcriptionally, or for which differences in transcript abundance that are undetectable on the array have large phenotypic consequences, contribute to the first source of discordance between the two methods. The second source of discordance could arise if the genes exhibiting expression changes during starvation are truly candidate genes affecting starvation resistance, but the particular *P*-element insertional mutation tested was not in a region affecting the starvation phenotype; a *P*-element insertion or point mutation in another location might produce a significant effect on starvation tolerance [45]. Another possibility is that the gene is downregulated during starvation; thus, a *P*-element mutation in the gene might not have an effect on the starvation resistance phenotype. Alternatively, a fraction of these probe sets could be false positives. Therefore, we conclude that assessing the effects of mutations at genes exhibiting changes in transcript abundance in response to an environmental (or genetic [5,7]) perturbation is a highly efficient strategy for identifying networks of pleiotropic genes regulating complex traits.

### Genetic variation in transcript abundance and quantitative trait phenotypes

The prospects for easily identifying genes corresponding to QTLs using microarray profiling seem less rosy at present. It has been proposed that candidate genes corresponding to QTLs are those for which expression differs between the parental strains used to construct the QTL mapping population, and which are located in the regions to which the QTLs map [20]. However, differences in expression between lines could be due to polymorphisms between the tested strains and the strain used to construct the probe sets on the array. Further, the lines differ for many traits, and QTLs affecting them overlap; unless the QTLs are mapped with very high resolution, candidate genes chosen by this criterion alone could affect another trait. The issue of polymorphism can be circumvented for traits with environmentally conditional expression by considering probe sets exhibiting a line × treatment environment interaction, and trait specificity can be addressed by correlating expression levels with the trait phenotype. None of these criteria led to an enrichment of candidate genes with variation in expression within QTL regions.

A major difficulty in using changes in gene expression between two strains to identify candidate genes corresponding to QTLs arises because variation in transcript abundance for positional candidate genes could arise from several causes. First, variation in transcript abundance is attributable to regulatory polymorphism in the candidate gene itself. Second, the candidate gene is itself not genetically variable, but regulatory variation in a second gene affects variation in its expression. Third, variation in transcript abundance at the candidate gene is attributable to interacting regulatory polymorphisms in both the candidate gene and a second gene. These interactions could be additive or epistatic. Positional candidate genes with variation in transcript abundance arising from the first or third cause could potentially correspond to genetically variable QTLs. However, it is becoming clear that genetic variation in transcript abundance is largely attributable to regulation by unlinked genes (see [15-17,19] and this paper). Indeed, single *P*-element insertions can alter the transcript expression of as many as 161 genes compared to a co-isogenic control line [5]. This low signal-to-noise ratio means that choosing positional candidate genes for further study based only on differences in transcript abundance between parental lines does not have a high likelihood of success.

In the future, the falling cost of whole-genome expression analysis will facilitate assessing transcriptional variation and variation in trait phenotypes in the same large QTL mapping populations. Co-localization of QTLs with main effects jointly affecting variation in transcription and trait phenotype will help winnow out monomorphic genes that are regulated by unlinked loci, and such data would enable direct tests for epistasis at the level of transcription and the trait. It is unlikely that this approach will completely supplant high-resolution QTL mapping and complementation tests to mutations for elucidating the genetic architecture of complex traits in *Drosophila*. None of the 12 candidate genes affecting variation in starvation resistance between Ore and 2b [21] exhibited variation in transcript abundance in this study. Possibly any transcriptional differences between Ore and 2b alleles at these loci are rare messages below the threshold of detection, or that are expressed in only a few cell types or at a particular period of development. In addition, not all allelic differences between QTL alleles are necessarily regulated at the level of transcription. Nevertheless, incorporation of knowledge about variation in transcript abundance will greatly inform our choice of candidate genes for confirmation by mutant complementation tests and association studies, which is currently biased by our poor understanding of the pleiotropic and epistatic consequences of variation in positional candidate genes on variation in trait phenotypes.

## Materials and methods

### *Drosophila *stocks

We used the isogenic lines 2b [22,46] and Oregon-R [47] (Ore) to establish 98 RI lines for mapping QTLs affecting starvation resistance [21]. Survival times for Oregon-R flies were 36.0 and 51.6 h for males and females, respectively. For 2b, survival times were 29.2 h for males and 40.4 h for females. Here, we assessed transcriptional profiles under control conditions and during starvation for 2b, Ore, two starvation resistant (RI.14, RI.21) and two starvation sensitive (RI.35, RI.42) RI lines. Recombination breakpoints for the RI lines have been determined previously [23] and are resolved to the nearest cytological lettered subdivision. We maintained control flies on cornmeal-agar-molasses medium, and starved flies on non-nutritive (1.5% agar and water) medium, under standard culture conditions (25°C, 70% humidity, and a 12-h light: 12-h dark cycle).

### Starvation half-life

We assessed survival of all six lines under starvation conditions by placing two replicates of ten flies each per sex on starvation medium, and recording the number of flies alive at 8-h intervals until all were dead. We used these survival curves to infer the starvation half-life for each line/sex combination. We used an analysis of variance (ANOVA) model *Y *= *μ* + *L *+ *S *+ *L *× *S *+ *R*(*L *× *S*) + *E*, to partition variance in survival times into sources attributable to the cross-classified main effects of lines (*L*), sex (*S*), variance between replicate vials (*R*), and within-vial environmental variance (*E*).

### Transcriptional profiling

For each of two independent replicates, we collected 300 male and 300 female virgins from all lines, aged 2-5 days post-eclosion. The control treatment consisted of 100 non-starved flies/line/sex. We placed the remaining 200 flies/line/sex on starvation medium, and collected approximately 100 flies/line/sex at the predetermined starvation half-life. Starved flies from all lines should therefore be in roughly the same physiological condition. We extracted whole-body RNA from each of the 48 independent samples (6 lines × 2 treatments × 2 sexes × 2 replicates) with Triazol reagent (Gibco BRL), followed by DNase digestion (RQ1 DNase, Promega,) and a 1:1 phenol (Sigma-Aldrich)-chloroform (Fisher Scientific) extraction. We hybridized biotinylated cRNA probes to single-color whole-genome Affymetrix Drosophila GeneChip arrays as described in the Affymetrix GeneChip Expression Analysis 2000 manual.

### Data analysis

We normalized the expression data by scaling overall probe set intensity to 100 on each chip using standard reference probe sets on each chip for the normalization procedure. Each probe set on the array consists of 14 perfect match (PM) and single nucleotide mismatch (MM) pairs. We used the average difference (AD) in normalized RNA expression between the 14 perfect match (PM) and mismatch (MM) probe pairs per probe set (Affymetrix Microarray Suite, Version 4.0) as the analysis variable. We calculated the minimum AD threshold value [5] as AD = 30. If the mean AD of a probe set was less than 30, and the maximum AD value was also less than 30, we eliminated the probe set from further consideration. We set all remaining AD scores < 30, to AD = 30. We performed a three-way factorial ANOVA of AD for each probe set, according to the model: *Y *= *μ* + *S *+ *T *+ *L *+ *S *× *T *+ *S *× *L *+ *T *×*L *+ *S *× *T *× *L *+ *E*, where *S*, *T*, and *L *represent, respectively, the fixed cross-classified effects of sex, treatment (control versus starved), and line, and *E *is the replicate variance between arrays. We determined F-ratio tests of significance for each term in the ANOVA, and considered probe sets with *P *values ≤ 0.001 for any term to be significant. (There are approximately 14,000 probe sets on the array; thus 14 false positives would be expected at this significance threshold.)

We computed the female:male ratio of AD values, averaged over all lines and treatments, for probe sets for which the main effect of *S *was significant. Similarly, we computed the starved:control ratio of AD values, averaged over lines and sex, for probe sets with significant *T *terms. We categorized these probe sets according to their gene ontology (GO) for biological process and molecular function [48]. We assessed significant differences in GO categories between up- and downregulated probe sets using *G *tests [49], under the null hypothesis of equal numbers of up- and down- regulated probe sets in each category, and using Bonferroni corrections to account for multiple tests. For probe sets with significant *T *× *S *terms, we ran two-way ANOVAs separately by sex using the reduced model *Y *= *μ* + *L *+ *T *+ *L *× *T *+ *E*.

Probe sets with significant *L*, *L *× *S*, *L *× *T *or *L *× *T *× *S *terms are candidate QTLs for traits that vary among the lines. We performed *post-hoc *Tukey tests for all probe sets for which these terms were significant to determine in which lines transcription was up-or downregulated in response to starvation stress. For probe sets that were significant for the main effect of *L*, but not any of the interaction terms, we conducted Tukey tests using the expression values pooled across control and starved conditions and both sexes. We computed Tukey tests separately for males and females, averaged over both treatments, for probe sets that were significant for the *L *× *S *interaction; and separately by treatment, for probe sets significant for the *L *× *T *interaction. The Tukey analyses separated the lines into groups within which AD values were not significantly different. Since the genotype for each recombinant inbred line at any given location is known, we used the Tukey analyses to classify probe sets as exhibiting linked or unlinked regulation of transcript abundance. We considered linked factors to regulate transcript abundance if Ore and 2b differ in transcript abundance, and this difference is reflected in the RI lines according to their Ore and 2b genotype in the region to which the gene maps. Conversely, we inferred that unlinked factors regulate transcript abundance in cases where there is not a 1:1 correspondence between parental line genotype and Tukey grouping. We determined the fold-change between Tukey groupings by calculating the ratio of the deviant line(s) expression level to the mean expression level of the parental or common group. Most Tukey analyses were unambiguous; where multiple interpretations were possible, we calculated the fold-change for all possibilities.

### Statistical analyses

We used SAS procedures for all statistical analyses [50].

## Additional data files

The following additional data files are available with the online version of this article. Additional data file 1 contains a list of all probe sets with significantly different expression in females and males. Additional data file 2 contains a list of all probe sets with significantly different expression under control and starved conditions. Additional data file 3 lists the probe sets for which the sex by treatment interaction term is significant. Additional data file 4 shows the correspondence between the results of a screen for the effects on resistance to starvation stress for single *P*-element inserts, in a co-isogenic background [21], and changes in transcript abundance between control and starved treatments. Additional data file 5 summarizes probe sets for which there is significant genetic variation in transcript abundance. Additional data file 6 shows the probe sets for which the only significant genetic term was the main effect of line. Additional data file 7 gives the same information as Additional data file 6, but separately for the control and starved treatments, and with the results of the analyses pooled over sexes, and for males and females separately. Additional data file 8 is the ANOVA of starvation half-life for Ore, 2b, RI.14, RI.21, RI.35 and RI.42. Additional data files 9 and 10 give the raw expression data and presence/absence calls for the control and starved treatments, respectively.

## Supplementary Material

Additional File 1 The file includes all *P*-values from ANOVA of expression values; the mean expression in males and females, averaged across treatments and lines; the FlyBase ID, gene name, symbol and synonyms; cytological location; and molecular function, biological process and cellular component gene ontologies Click here for file

Additional File 2 The file includes the mean expression levels under control and starved conditions, averaged over sexes and lines, as well as the information given for each probe set in Additional data file 1 Click here for file

Additional File 3 The file includes the *P*-values for the treatment term in the reduced analyses for males and females separately; the mean expression values under control and starved conditions for males and females, averaged over lines; whether expression is sex-biased (SB), sex-specific (SS) or sex-antagonistic (SA); plus the information given for each probe set in additional data file 1 Click here for file

Additional File 4 The results are grouped into four categories: (1) Transcript abundance is altered between control and starved treatments, and there is a significant effect of the *P*-element insertion on starvation tolerance; (2) Transcript abundance is altered between control and starved treatments, but the *P*-element insertion does not significantly affect starvation tolerance; (3) There is no alteration in transcript abundance between control and starved treatments, but the *P*-element insertion significantly affects starvation tolerance; and (4) There is no alteration in transcript abundance between control and starved treatments, and no significant effect of the *P*-element insertion on starvation tolerance Click here for file

Additional File 5 The file includes all *P*-values from ANOVA of expression values; the gene name, symbol, synonyms, and cytological location; the molecular function, biological process and cellular location gene ontologies; co-localization of the gene with QTLs affecting life span, sensory bristle numbers, starvation resistance, ovariole number, courtship signal, flight, metabolic rate and triglycerides; and the statistical association of variation among lines in transcript levels and starvation half-life Click here for file

Additional File 6 The cells are color-coded: purple for Ore genotype, green for 2b genotype, gray if the genotype is unknown because the gene is between an Ore and a 2b flanking marker, and the exact recombination breakpoint is not determined, and gold if the genotype is unknown because of residual heterozygosity in the RI line. The results of Tukey tests separating the lines into groups within which expression values are not significantly different are given. If more than one interpretation of the Tukey groups are possible, all are given. Linked (L) regulation of variation in transcript abundance is inferred if the Tukey groupings match the genotype. Regulation of variation in transcript abundance is inferred to be linked (IL) if the unknown genotypes could match this interpretation. Unlinked (U) regulation of variation in transcript abundance is inferred if the Tukey groupings do not match the line genotypes Click here for file

Additional File 7 A table giving the same information as in Additional data file 6, but separately for the control and starved treatments, and with the results of the analyses pooled over sexes, and for males and females separately Click here for file

Additional File 8A table showing the ANOVA of starvation half-life for Ore, 2b, RI.14, RI.21, RI.35 and RI.42Click here for file

Additional File 9 A table showing the raw expression data and presence/absence calls for the control treatments Click here for file

Additional File 10 A table showing the raw expression data and presence/absence calls for the starved treatments Click here for file

## Figures and Tables

**Table 1:** Gene Ontology categories with sex-biased gene expression

Gene Ontology category	Number of upregulated probe sets		*P*-value*
		
	Females	Males	
**Biological process**			
Cell communication			
Signal transduction	135	40	<0.0001
			
Cell growth and/or maintenance			
Cell cycle	184	15	< 0.0001
Cell organization and biogenesis	207	65	< 0.0001
Transport	123	49	< 0.0001
Biosynthesis	238	43	< 0.0001
Catabolism	71	24	< 0.0001
Nucleic acid metabolism	374	28	< 0.0001
Phosphorous metabolism	147	60	<0.0001
Protein metabolism	495	113	< 0.0001
			
Development			
Cell differentiation	33	11	7.41 × 10^-4^
Embryonic development	126	27	< 0.0001
Morphogenesis	200	50	< 0.0001
Pattern specification	76	9	<0.0001
Post-embryonic	50	11	< 0.0001
Gametogenesis	164	20	< 0.0001
Other development	84	17	< 0.0001
Cell death	25	5	1.54 × 10^-4^
			
**Molecular function**			
Binding			
DNA binding	310	46	< 0.0001
Nuclease	31	3	< 0.0001
RNA binding	180	38	< 0.0001
Translation factor	40	13	1.58 × 10^-4^
Nucleotide binding	187	68	< 0.0001
			
Protein binding			
Cytoskeletal protein binding	89	43	< 0.0001
Transcription factor binding	28	3	< 0.0001
			
Enzymes			
Hydrolase enzyme			
Acting on acid anhydrides	177	94	< 0.0001
Acting on ester bonds	113	56	< 0.0001
Kinase enzyme	156	62	< 0.0001
Ligase enzyme	52	18	< 0.0001
Oxidoreductase enzyme	69	139	< 0.0001
Transferase enzyme	327	105	< 0.0001
Other enzymes	88	16	< 0.0001
			
Signal transducer			
Signal transducer - receptor signaling protein	89	14	< 0.0001
Structural molecule			
Ribosome structure	137	8	< 0.0001
Transcription regulator	199	35	< 0.0001
Translation regulator	42	13	< 0.0001
			
Transporter			
Carrier transporter	82	143	< 0.0001
Ion transporter	30	70	< 0.0001

*Significant after Bonferroni correction.

**Table 2:** Gene Ontology categories with increased or decreased gene expression during starvation

Gene Ontology category	Number of probe sets		*P*-value*
		
	Upregulated	Downregulated	
**Biological process**			
Cell growth and/or maintenance			
Biosynthesis	119	31	< 0.0001
Protein metabolism	220	95	< 0.0001
Development	12	35	6.48 × 10^-4†^
Behavior	1	9	8.10 × 10^-3‡^
			
**Molecular function**			
Binding			
Nucleotide binding	76	38	3.36 × 10^-4^
Defense/immunity protein	3	18	6.55 × 10^-4^
			
Enzymes			
Hydrolase			
Acting on acid anhydrides	77	42	1.25 × 10^-3^
Peptidase	50	104	1.12 × 10^-5^
			
Structure			
Cuticle structure	1	14	3.09 × 10^-4^
Ribosome structure	84	3	< 0.0001
			
Transporter			
Carrier	46	84	8.05 × 10^-4^
Signal transducer	2	12	5.67 × 10^-3†^

*Significant after Bonferroni correction; ^†^significant for females only; ^‡^significant for males only.

**Table 3:** Association of genetic variation in transcription with genetic variation in quantitative traits

Trait	QTL^†^			Not QTL		χ^2^_1_
			
	Number	Probe sets^‡^	kb	Probe sets^‡^	kb	
Life span [22]	5	125	25,351	350	92,625	6.58*
Sternopleural bristle number [25]	5	250	54,150	225	63,853	8.70**
Abdominal bristle number [25]	7	154	34,038	321	83,965	2.96 NS
Starvation resistance [21]	5	110	26,532	365	91,471	0.12 NS
Life span [21]	4	98	24,305	377	93,698	0.00 NS
Life span [23]	4	133	32,899	342	85,104	0.00 NS
Ovariole number [26]	2	70	13,162	405	104,841	6.15*
Life span [24]	5	82	19,637	393	98,366	0.13 NS
Olfactory behavior [28]	1	36	7,944	439	110,059	0.54 NS
Courtship signal [27]	3	67	15,859	408	102,144	0.18 NS
Flight [29]	2	119	27,860	356	90,143	0.55 NS
Metabolic rate [29]	2	41	8,232	434	109,771	2.01 NS
Glycogen [29]	2	5	4,683	470	113,320	10.60 **^‡^
Triglycerides [29]	2	30	6,044	445	111,959	1.39 NS

^†^Two LOD support intervals. In cases of overlap of support intervals between adjacent QTLs, the two QTLs were merged into a single region spanning both. ^‡^*P*(line) and/or *P*(Sex × line) < 0.001. ^§^Significant after Bonferroni correction. ***P < 0.001; **0.001 <*P *< 0.01; *0.01 <*P *< 0.05; NS *P *> 0.05.

**Table 4:** Candidate QTLs for starvation resistance

Probe set	Significant*	Gene	Location	Molecular function	Biological process	Cellular location
151378	*S, L, r*	*mitochondrial ribosomal protein L33*	4B6	Structural constituent of ribosome	Protein biosynthesis	Mitochondrial large ribosomal subunit
151504	*L*	*no receptor potential A*	4C1	1-phosphatidylinositol-4,5-bisphosphate phosphodiesterase; phospholipase C	Olfaction; response to abiotic stimulus	inD signaling complex; membrane fraction; rhabdomere
153437	*S, T, L, r*	*yippee interacting protein 2*	30E4	Acetyl-CoA C-acyltransferase	Fatty acid beta oxidation	Mitochondrion
146142	*S, T, L, r*	*Selenophosphate synthetase 2*	31D9	Selenide, water dikinase; purine nucleotide binding	Selenocysteine biosynthesis	
143984	*S, T, S *× *T, L, L *× *S*	*Accessory gland-specific peptide 32CD*	32D1	Hormone	Negative regulation of female receptivity, post-mating	Extracellular
141745	*S, L, L *× *S, L *× *T, r*	*Phosphoethanolamine cytidylyltransferase*	34A9	Ethanolamine-phosphate cytidylyltransferase	ethanolamine and derivative metabolism; phospholipid metabolism	
146347	*S, L, L *× *S, L *× *T, L *× *S *× *T*	*centaurin gamma 1A*	34D6-E2	ARF GTPase activator	G-protein-coupled receptor protein signaling pathway; small GTPase mediated signal transduction	Nucleus
153741	*L *× *T*	*centaurin gamma 1A*	34D6-E2	ARF GTPase activator	G-protein coupled receptor protein signaling pathway; small GTPase mediated signal transduction	Nucleus
143402	*S, L, L *× *S, r*	*vasa*	35C1	RNA helicase activity; nucleic acid binding; ATP dependent helicase	Dorsal appendage formation; oogenesis; pole plasm RNA localization; pole plasm assembly	Polar granule
152721	*T, L, L *× *T, r*	*Imaginal disc growth factor 1*	36A1	Imaginal disc growth factor activity; NOT chitinase activity; hydrolase activity, hydrolyzing N-glycosyl compounds	Cell-cell signaling;signal transduction	Extracellular
154661	*S, L*	*midway*	36B1-2	Sterol O-acetyltransferase; diacylglycerol O-actyltransferase	Cholesterol metabolism; triacylglycerol biosynthesis	
152756	*S, L, r*	*Arrestin 1*	36D3	Metarhodopsin binding	G-protein coupled receptor protein signaling pathway; deactivation of rhodopsin mediated signaling; endocytosis; intracellular protein transport; metarhodopsin inactivation	Membrane fraction; rhabdomere
143876	*S, L*	*Galactose-specific C-type lectin*	37D6	Galactose binding; sugar binding; receptor	Defense response	
146555	*S, T, S *× *T, L, L× S*	*Serine protease inhibitor 3*	38F2	Serine-type endopeptidase inhibitor	Proteolysis and peptidolysis	
146592	*S, T, S *× *T, L× T, L× S× T*	*no mechanoreceptor potential B*	39E2		NOT flagellum biogenesis; perception of sound; sensory cilium biogenesis	
143709	*S, T, L, r*	*Troponin C at 41C*	41E5	Calcium ion binding; calmodulin binding	Calcium-mediated signaling; muscle contraction	
143127	*S, T, L, L *× *T*	*Cytochrome P450-6a2*	42C8-9	Electron transporter activity; oxidoreductase	Response to insecticide; steroid metabolism	Membrane; microsome
146718	*S, T, L *× *T*	*Tetraspanin 42Er*	42F1	Receptor signaling protein	Ectoderm development; neurogenesis; transmission of nerve impulse	Integral to membrane
142222	*T, L, L *× *T*	*Cytochrome P450-9b2*	42F3	Electron transporter activity; oxidoreductase		Membrane; microsome
143830	*S, L*	*Calcineurin B2*	43E16	Calmodulin binding; calcium-dependent protein serine/threonine phosphatase, regulator; calcium ion binding	Calcium-mediated signaling; cell homeostasis	Calcineurin complex
141501	*S, T, L, r*	*Proteasome alpha6 subunit*	43E18	Proteasome endopeptidase	Proteolysis and peptidolysis	20S core proteasome complex
143303	*S, T, L, r*	*photorepair*	43E18	Deoxyribodipyrimidine photolyase; nucleic acid binding	DNA repair	
146780	*S, L *× *T, L *× *S *× *T, r*	*Sep5*	43F8	Structural constituent of cytoskeleton; small monomeric GTPase	Cytokinesis; mitosis	Septin ring
143780	*L, L *× *S*	*Cytochrome P450-4e1*	44D1	Electron transporter activity; oxidoreductase		Membrane; microsome
152113	*S, T, L *× *S, r*	*anachronism*	45A1		Suppression of neuroblast proliferation	Extracellular
143554	*S, L*	*trp-like*	46B2	Calcium channel; calmodulin binding; light-activated voltage-gated calcium channel; store-operated calcium channel	Calcium ion transport	Plasma membrane; rhabdomere
146946	*S, T, L *× *T, r*	*Peroxiredoxin 2540*	47A7	Antioxidant; peroxidase; non-selenium glutathione peroxidase	Defense response; oxygen and reactive oxygen species metabolism	
143603	*T, L*	*gammaTrypsin*	47F4	NOT serine-type endopeptidase	Proteolysis and peptidolysis	Extracellular
143602	*T, L*	*betaTrypsin*	47F4	Trypsin	Proteolysis and peptidolysis	Extracellular
143604	*T, L*	*gammaTrypsin*	47F4	NOT serine-type endopeptidase	Proteolysis and peptidolysis	Extracellular
143624	*T, L *× *T*	*epsilonTrypsin*	47F4	Trypsin	Proteolysis and peptidolysis	Extracellular
153279	*S, T, L, r*	*Translocon-associated protein d*	47F7	Signal sequence receptor	Protein-ER retention	Signal sequence receptor complex; translocon
141563	*L*	*acyl-Coenzyme A oxidase at 57D proximal*	57E1	Acyl-CoA oxidase; palmitoyl-CoA oxidase	Fatty acid beta-oxidation	Peroxisome
151902	*S, T, L, r*	*jitterbug*	59A3	Actin binding; structural constituent of cytoskeleton	Cytoskeleton organization and biogenesis	
154177	*S, L, L *× *S, r*	*Cyclin B*	59B2	Cyclin-dependent protein kinase, regulator	Cytokinesis; mitotic anaphase B; mitotic chromosome movement	Nuclear cyclin-dependent protein kinase holoenzyme complex; pole plasm
143203	*S, T, L, r*	*inactivation no afterpotential D*	59B3	Structural molecule; calmodulin binding; myosin binding; receptor signaling complex scaffold	Cell surface receptor linked signal transduction; phototransduction; protein targeting	inaD signaling complex; rhabdomere
151517	*L*	*Phosphotidylinositol 3 kinase 59F*	59E4-F1	Phosphatidylinositol 3-kinase; phosphoinositide 3-kinase	Endocytosis; phosphoinositide phosphorylation; protein targeting	Phosphoinositide 3-kinase complex, class III
151830	*S, T, L, L *× *T, r*	*lethal (2) essential for life*	59F6	Heat shock protein	Defense response; protein folding; response to stress	
144140	*T, L, r*	*Mitochondrial phosphate carrier protein*	70E1	Phosphate transporter; carrier	Phosphate metabolism; phosphate transport	Mitochondrial inner membrane
151748	*L, L *× *T, r*	*Cyclic-AMP response element binding protein A*	71E1	DNA binding; RNA polymerase II transcription factor; transcription factor	Salivary gland morphogenesis; transcription from Pol II promoter	Nucleus
153226	*S, T, L*	*Argonaute 2*	71E1	Translation initiation factor; protein binding	RNA interference; translational initiation	RNA-induced silencing complex

*Significant (*P *< 0.001) for the main effects of Sex (*S*), treatment (*T*), line (*L*) and their interactions from ANOVA of transcript abundance; significant (*P *< 0.05) correlation (*r*) between starvation half-life and transcript abundance.
