# Supplementary material for: Quantitative genomics of starvation stress resistance in Drosophila
Source: Genome Biol. 2005 Mar 24;6(4):R36. doi: 10.1186/gb-2005-6-4-r36 (PMC1088964; doi:10.1186/gb-2005-6-4-r36)
Supplement: Additional File 8 — A table showing the ANOVA of starvation half-life for Ore, 2b, RI.14, RI.21, RI.35 and RI.42 [file gb-2005-6-4-r36-S8.doc]

Analysis of variance of starvation half-life for Ore, 2b, RI.14, RI.21, RI.35Y and RI.42Y

|  | Source | df | MS | *F* | *P* | σ^2^ |
| --- | --- | --- | --- | --- | --- | --- |
| Sexes pooled | Sex | 1 | 5860.1 | 47.1 | < 0.0001 | - |
|  | Line | 5 | 9525.6 | 76.5 | < 0.0001 | 131.5 |
|  | Line×Sex | 5 | 600.2 | 4.8 | 0.0120 | 23.8 |
|  | Rep(Line×Sex) | 12 | 124.5 | 1.0 | 0.4072 | 0.6 |
|  | Error | 216 | 119.0 | - | - | 119.0 |
| Males separately | Line | 5 | 1537.8 | 11.1 | 0.0054 | 70.0 |
|  | Rep(Line) | 6 | 138.1 | 1.9 | 0.0868 | 6.6 |
|  | Error | 108 | 72.6 | - | - | 72.6 |
| Females separately | Line | 5 | 4922.5 | 44.4 | < 0.0001 | 240.6 |
|  | Rep(Line) | 6 | 110.9 | 0.7 | 0.6733 | 0.0 |
|  | Error | 108 | 165.3 | - | - | 165.3 |
